# Why Are Spanish Nurses Going to Work Sick? Questionnaire for the Measurement of Presenteeism in Nurses

**DOI:** 10.3390/nursrep11020032

**Published:** 2021-05-06

**Authors:** Carmen María Sarabia-Cobo, María Sáenz-Jalón, Pedro Cabeza-Díaz, Blanca Torres-Manrique, Obdulio Manuel González-Martínez, Esperanza Alonso-Jiménez, David Cantarero-Prieto, Marta Pascual-Sáez

**Affiliations:** 1 Facultad de Enfermería, Universidad de Cantabria, IDIVAL, 39011 Santander, Spain; 2 Hospital 12 de Octubre, IDIVAL, 28050 Madrid, Spain; mariasaenzjalon@gmail.com; 3 Solvay Química, SL.L., 39300 Barreda, Spain; pcabezad@gmail.com; 4 Servicio Cántabro de Salud, 39011 Santander, Spain; blanca.torres@unican.es (B.T.-M.); dulio@eresmas.com (O.M.G.-M.); 5 Servicio de Prevención, 33011 Asturias, Spain; emariaaj@gmail.com; 6 Facultad de Ciencias Económicas y Empresariales, Universidad de Cantabria, 39011 Santander, Spain; david.cantarero@unican.es (D.C.-P.); marta.pascual@unican.es (M.P.-S.)

**Keywords:** absenteeism, burnout, nursing, presenteeism, work, safety

## Abstract

Presenteeism is defined as the presence of the worker at their workplace despite not being in optimal physical or mental conditions. Presenteeism is a phenomenon that has been poorly studied in the context of healthcare. Despite the many negative consequences associated with presenteeism, to date, no studies have investigated this issue in nurses in Spain. The objective was to develop and validate a questionnaire on presenteeism to be used by nursing staff in Spain. Methods: A psychometric study for the development and validation of a questionnaire. The PRESENCA^®^ questionnaire on presenteeism was created by a panel of experts, based on a survey comprised of 31 Likert-type items. Results: In total, 355 nurses completed the questionnaire. The factorial analysis revealed the existence of 3 factors and confirmed appropriate levels of validity and reliability (alpha = 0.729). Conclusions: The PRESENCA^®^ questionnaire is the first tool developed and validated in Spanish for the assessment of presenteeism in nursing. Our findings demonstrate that this scale has appropriate psychometric properties and its use may facilitate the detection of presenteeism among professionals. As a result, use of this questionnaire may contribute towards the improvement of clinical safety.

## 1. Introduction

Presenteeism in workplaces is a much costlier problem than other direct or indirect social and economic burdens. Presenteeism has been defined as productivity loss due to health problems or other events that adversely affect employees.

Research on work absenteeism constitutes an important indicator for the management and estimation of professional resources. However, in recent years, another phenomenon has raised interest: presenteeism [1]. Presenteeism is defined as the presence of the worker at their workplace despite not being in optimal physical or mental condition [2]. This has a number of consequences, such as decreased productivity and economic losses. Presenteeism should not be confused with absenteeism, which is voluntary nonattendance at work, without valid reason [1,2]. Interestingly, presenteeism is a phenomenon that has been poorly studied in the context of healthcare. Most studies published on the subject to date have attempted to understand the reasons that lead professionals to go to work despite being in suboptimal physical or mental health conditions and knowing they will be unable to perform to the best of their abilities [3].

The recent interest in presenteeism began in the middle of the last decade. Since then, the number of studies on this subject has increased considerably, especially since the publication of an article in the Harvard Business Review in 2004, which indicated that the yearly losses attributed to presenteeism may exceed 150 billion dollars per year in the United States of America (USA), surpassing the losses caused by days off taken by workers who fall ill [4]. Some experts consider that the effects of presenteeism are more harmful and the costs are higher than those caused by absenteeism, as workers in the former situation go to work and, besides not performing their activity, they are paid a complete wage. Some estimates suggest that up to 72% of productivity losses may be due to presenteeism compared to 28% as a result of days off because of illness [5].

It is difficult to measure the economic impact of presenteeism as, in many professions such as nursing, the product of the job is difficult to accurately measure, define and quantify in terms of its cost. However, a study performed in North Carolina (USA) reported that the approximate economic costs of presenteeism in nursing were close to 22 billion dollars [6]. Another disadvantage when trying to measure the economic impact of presenteeism is that the repercussion this has on the rest of the team [greater workloads for colleagues, etc.] and on the company at large should also be considered [7].

Presenteeism must not be confused with workers performing unproductive tasks at work [i.e., drinking coffee, going out to smoke], or with those who simulate an illness in an attempt to avoid performing certain tasks [8,9].

The illnesses which are most commonly associated with presenteeism tend to be of a venial nature, including allergies, flu, migraine, low back pain, sinusitis, asthma, stress or depressive states, and which are not accepted by workers as sufficiently incapacitating, or as affecting their competence to perform their work [3,10]. Health problems are also associated with work productivity, as work affects health and health affects work [11]. Several studies have shown that two thirds of people with depression are untreatable and the probability of suffering an accident at the workplace for those who go to work with a physical, mental or emotional deficit is higher compared to people who do not suffer from these disorders, as well as increasing the possibility of errors [12,13]. Furthermore, whereas depression and anxiety may be the most common disorders in the workplace, these illnesses are often more common in presenteeism (and related to a decrease of productivity) than absenteeism [1,14,15,16,17].

The studies performed on this phenomenon to date show that nursing is one of the professions with the highest presenteeism, several researches concluded that the rates of presenteeism were four times higher in nursing professionals compared to other employees [1,3,5]. Furthermore, current studies suggest that the poor health and wellbeing of health professionals may have repercussions on clinical safety and the quality of care provided [7,18]. Other studies also point out that stress, long workdays and the effects of staff cuts on the ability for nurses to provide the specific care required by patients can lead to reduced productivity rates and significant numbers of nurses contemplating abandoning the profession [8,19].

According to the literature, several reasons may explain why nurses go to work even though they know they are not in the best physical or mental conditions to do so, such as high work demands, not having appropriate coverage/replacement available, workplace insecurity, feelings of stress at the workplace, and so on [1,2,13,20,21].

On account of all the above reasons, many nurses decide to go to work despite not being at 100% of their capacity and, hence, there may be a relation between presenteeism and reduced patient safety (although no studies have explicitly related these issues). This is because the errors that are made in healthcare frequently involve professionals who are in direct contact with the patient, where physiological problems such as lack of sleep, fatigue, suffering from some kind of illness (acute or chronic), and/or taking medication, bear a relation with the errors made [22,23].

Several questionnaires have been developed and validated to assess the causes, characteristics and consequences of presentiment in nurses [24,25,26], and the most well-known and internationally translated is the questionnaire developed by the University of Stanford [24]. In Spain, several previous studies have been carried out, some of them comparative among different countries, with interesting results [27,28]. However, they used the Stanford questionnaire, which is especially suited to nurses working in the private sector and has few items, so its sensitivity is not very high. Although the results are valid, we have carried out the creation and validation of a questionnaire specific to the reality of the Spanish healthcare system (similar to others in Europe) aimed not only at nurses working in hospitals, but also at nurses in all healthcare settings (something very rare in the literature reviewed) [1,2,3,4,5,6,7,29]. The results will allow us to detect differences in terms of causes and consequences depending on the place and working conditions of the nurses. This may facilitate decision-making to avoid and prevent this situation, which is becoming increasingly frequent as we have seen with the current coronavirus disease 2019 (COVID-19) pandemic.

The aim of our study was the development and validation of a questionnaire to measure presenteeism among nursing professionals in Spain.

## 2. Methods

A psychometric study for the development and validation of a questionnaire. A cross-sectional descriptive study.

Subjects were nursing professionals registered in the Cantabria Official Nursing Board, N = 3000 nurses. The only inclusion criterion was the acceptance to participate in the study.

### 2.1. Variables

Independent variables: age, sex, qualification, specialty, place of work (public/private, hospital/primary care/mutual insurance/public health center), work situation, work experience.

Dependent variables. Rate of presenteeism measured using the so-called PRESENCA^®^ scale for the measurement of presenteeism.

### 2.2. Questionnaire Development Procedure

The PRESENCA^®^ questionnaire was created by a panel of experts comprising four nurses who were specialized in occupational nursing and who independently created a preliminary questionnaire.

The content validity of the questionnaire was analyzed based on the criteria of 20 experts. These were specialists in occupational nursing and included nationwide representatives belonging to the Spanish Federation of Occupational Nursing (FEDEET). Representative sample sizes were used and a non-parametric test (Kendall’s W) was used to test the level of agreement between experts.

The Delphi method was used as well as the Kendall’s concordance test. Brainstorming sessions took place in which the selected experts proposed a series of characteristics or attributes which they considered must form part of the questionnaire. Furthermore, they had to respond to an ad hoc survey designed to evaluate the questionnaire. This step was performed twice, which led to a series of suggestions and changes. This resulted in 23 questions, with Likert-type response options. The scale measures the participant’s concentration skills and work performance skills, despite having a health problem, via two factors: finalizing the task and maintaining concentration. The first factor is related to physical aspects and the second refers to psychological issues.

Subsequently, permission was sought from the relevant Clinical Research Ethics Committee to perform a pilot test using the version agreed upon by the panel of experts. The pilot test was conducted with 50 professionals, who were asked to evaluate: the level of understanding of the questions, whether the response scale was appropriate, detect any rejection towards any of the questions and whether the time needed to complete the survey was acceptable. The questionnaire was administered in paper format by the research team and the suggestions were gathered by the professionals included in the pilot test. Based on these changes, the final version of the questionnaire was created. 

The final version of the questionnaire consisted of 31 multiple choice questions (or Likert-type scale or dichotomous response).

### 2.3. Data Collection and Analysis

The questionnaire was sent to respondents online using the Google Docs Forms tool, via the Cantabria Board of Nursing, which sent an email with a link to access the questionnaire and which posted the link on its webpage during an agreed time period. A study information letter was attached to this link, together with instructions for completion of the questionnaire and a deadline for returning the same. The data were directly posted onto an Excel spreadsheet.

### 2.4. Ethical Considerations and Authorizations 

The project was approved by the Ethics Committee of the Cantabrian Health Service and all collected data has been treated according to the current legislation (code 2020.022).

### 2.5. Analysis of the Validity and Reliability of the Questionnaire

The construct validity was evaluated via the technique of exploratory factorial analysis of the main components and application of the solution via the Varimax method. Each variable was included in a single factor, according to its factorial load, establishing values of 0.50 as a minimum saturation criterion. Varimax rotation was assumed to be the most appropriate to discriminate the maximum factors that conform the scale. The Kaiser-Meyer-Olkin measure of sampling adequacy (KMO) was calculated (range between 0–1) and the Bartlett statistical significance (if the value is close to the unit and they are significant at *p* < 0.05, this indicates that the analysis with reduction of variables is appropriate). To analyze reliability, an evaluation of the internal consistency was performed using the Cronbach’s alpha coefficient, for the questionnaire total and for each of the independent factors, also, the item-total correlation was calculated and the Cronbach’s alpha value if item deleted. The data obtained were analyzed using the SPSS version 22.0 statistical software program in its Spanish version for Windows. The null hypothesis was rejected with a statistical significance of *p* < 0.05. A descriptive analysis was performed of frequencies and graphs according to the following variables: the qualitative variables were described according to the frequency and percentage distribution (for a confidence interval (CI) of 95%); the quantitative variables were described via the mean and the standard deviation. Hypothesis contrast tests were used (Student’s *t*-test and Chi-squared test, etc.) to compare the behavior of the variables, whereas the correlation of variables was evaluated using the Pearson’s *r* coefficient.

## 3. Results

In total, 355 completed questionnaires were collected (11.88% response rate). All the questions were answered by 100% of the professionals participating in this study.

### 3.1. Analysis of the Reliability and Validity of the Questionnaire

The Kaiser–Meyer–Olkin measure of sampling adequacy was calculated (*KMO* = 0.283) as well as the Bartlett statistical significance (*p* = 0.000). The commonality analysis regarding the specific presenteeism items (9 items) indicated that all the items were above 0.5, therefore all the questions were accepted as valid for inclusion on the questionnaire (see Table 1).

Employing the method of the main components, 3 components or main factors were obtained with values greater than 1, explaining 80.63% of the total explained variance, a percentage of explanation which is within the appropriate level of acceptance (see Table 2), highlighting Factor 1, with greater loading in the explained variance.

Each item was included in a single factor, according to its factorial load, establishing values of 0.50 as a minimum saturation criteria. The rotated factorial solutions according to Varimax conformed a well-defined structure without overlaps. Table 3 shows the items of the scale with their respective factorial loadings in each of the 3 factors.

Factor 1 (Sick leave) comprised the items Sick leave due to illness, Reason and Pathologies. Factor 2 (factors that are external to the worker) comprised Performance, Superiors and Colleagues, whereas factor 3 (presenteeism factors) comprised of Number of times, Adverse effects and Decreased faculties.

To evaluate the internal consistency of the questionnaire, a Cronbach’s alpha value of 0.729 was obtained. This value did not improve after eliminating some of these items, and therefore all items were maintained. The values of the item-total correlation ranged between 0.52 and 0.73.

As a result of the validation process, a final questionnaire was obtained, comprising 31 items, which determined 3 factors.

### 3.2. Analysis

In total, we gathered 355 completed questionnaires. The main sociodemographic and work-related variables of participants are displayed in Table 4.

Regarding the question “Over the last 12 months, have you been on sick leave?” 79.3% responded “No” and 20.7% responded “Yes”. In the case of the question “Over the last 12 months, while at work, have you ever had to absent yourself from duty because you weren’t feeling well, health-wise?” 78.4% answered “No” and 21.6% answered “Yes”. Upon asking the percentage of people who had been absent, the number of times this had occurred over the last 12 months, the findings were contradictory, as 52.3% responded “never” (*n* = 81), 31% responded “once” (*n* = 48), 15.5% responded “2–5 times” 15.5% (*n* = 24) and 1.3% responded “more than 5 times” (*n* = 2).

In response to: “Over the last 12 months, have you gone to work despite not being in full possession (100%) of your faculties?” 80.4% responded “Yes” and 19.3% responded “No”. When asked “Do you think that under these circumstances you are able to perform to 100% at your work post?” 80.7% responded “No”, 9.7% responded “Yes”, and 9.7% responded “I don’t know”.

When asked: “In your opinion, can going to work unwell increase the adverse effects or errors related to your care duty?” 85.5% responded “Yes” and 7.7% responded “No”.

Regarding the most common processes with which they went to work, these were gastrointestinal problems (16.2%), fever (8.2%), musculoskeletal problems (8.2%), allergies (5.4%), depression/anxiety (4%) and fever (2.3%).

In response to the question “Do you think that your presence at work despite feeling unwell is well looked upon by your superiors?” 81% responded “Yes” and 12.5% responded “No”. When asked: “Do you think that your presence at work despite feeling unwell is well looked upon by your colleagues?” 27% responded “Yes” and 11.9% responded “No”.

When asked: “In the case of having gone to work unwell, please indicate the motive or motives for going to work”, the main responses were: “I cannot miss work (41.5%, *n* = 146), “I lose money” (29.5%, *n* = 104), “Job instability” (25.3%, *n* = 89), “I cannot fail my patients” (23%, *n* = 81), “Not enough staff to cover substitutions” (18.2%, *n* = 64), “Camaraderie” (13.4%, *n* = 47).

When statistical significance testing was performed to establish the possible differences between the grouped variables we found:

› Upon comparing by type of workplace (Public/Private), no significant differences were found for any of the relevant questions concerning presenteeism: missing work because of illness (t = 1.43, *p* = 0.239), working without feeling 100% (t = 0.36, *p* = 0.691), believing that one can perform at 100% despite feeling ill (t = 1.38, *p* = 0.261), believing that when one is ill, errors are made at work (t = 1.47, *p* = 0.231).
› Upon comparing the previous questions by 10-year age groups, no significant differences were found.
› Neither did we find significant differences for the main questions on presenteeism upon comparing by type of contract (stable employment contract = indefinite + interim, temporary contract = fixed − term).

Regarding the profile of the employees suffering presenteeism, we considered this was the case of those who had to absent themselves from duty between two and five times over the past 12 months, due to feeling ill (15.5%, *n* = 24). The mean age of these participants was 41.19 years, 88.7% were women, 48.1% were single, 77% worked in companies with over 1000 workers, 64% held a Diploma in Nursing, 37.7% had over 20 years of experience, 80.2% had no specialty and for those who did, the most frequent was that of working (8.5%). Additionally, 97.9% were working at the time of the survey, 89.4% were working at a public center, 68.6% were working in a public hospital and 52.3% working at a specialized facility. Up to 44.9% had a fixed shift, whereas 44.5% had a rotating shift. Also, 49.3% were fixed staff and 88% were not moonlighting (holding multiple jobs). Additionally, 86.2% agreed to being able to perform to 100% at work despite not feeling well. However, 86.2% said that feeling unwell at work could increase the number of errors made during care duties. The main reason for working despite feeling unwell were gastrointestinal problems (18.1%); 78.1% reported that going to work despite feeling unwell was well looked upon by their supervisors, and 28.6% felt it was well looked upon by their colleagues. The main reasons for going to work despite feeling unwell were: fear of losing money (32.5%), job instability (26.5%), wanting to be there for their patients (23%) and because of a shortage of staff for covering substitutions (19.4%).

Regarding the study of possible correlations between the profile of the person displaying presenteeism and other variables, we found the following significant correlations:

A weak correlation between absenting themselves from work because of feeling ill and taking sick leave (rho s = 0.123, *p* = 0.021), interpretation: this is a striking finding, which, although weak, indicates that a greater number of sick leave days took place when employees had to absent themselves from work.

A strong negative correlation was found between having to absent themselves from work and the number of times they did so (rho s = −0.853, *p* = 0.000), (interpretation: a striking finding, suggesting that, on many occasions, when the person felt unwell enough to need to go home, they did not actually do so, and therefore, this is an indicator of presenteeism.

A weak, but positive correlation was found between having to absent themselves from work and working despite not being at 100% (rho s = 0.164, *p* = 0.002).

A weak negative correlation was found between going to work despite not feeling 100% and the number of times the person had to absent themselves from work (rho s = −0.200, *p* = 0.012). This interpretation is along the same lines as what we found regarding having to absent themselves and the number of times the participants actually did so.

A weak negative correlation was found between the type of center and work experience (rho s = −0.223, *p* = 0.00).

### 3.3. Binary Logistic Regression Model

A significant association was found between having to absent themselves because they were ill and the number of times the person had to absent themselves i.e., go home because they were ill, (odds ratio (OR) = 3.0, 95% CI = 1.6–5.6 (*p* < 0.00). Therefore, those who felt unwell while working had a threefold greater risk of having to leave work than those who did not. Also, there was a significant association between being in active employment (OR = 0.865, 95% CI = 0.6–1.4 (*p* < 0.00), and moonlighting (OR = 1.137, 95% CI = 0.9–1.9 (*p* = 0.033), which appears obvious.

## 4. Discussion

The aim of this study was to develop and validate an appropriate questionnaire for the detection and evaluation of presenteeism among nurses. Our findings have highlighted that the PRESENCA questionnaire has a good factorial structure, with a good predictive capacity and high internal consistency.

Our findings are in line with previous research that identifies the profile of the nurse presentism as a middle-aged woman, working in a large hospital, with many years of experience, a permanent position, and a strong sense of belonging to her unit or team [9,10,27]. We could say, according to our results, that age, working in a public hospital, having many years of experience and being a specialist can be good predictors of presenteeism [30,31].

The nurses in the study indicated that even though they knew they were not at 100%, it was more prevalent for them to go to work sick, for reasons such as the high workload, not leaving their colleagues alone, low staffing levels, etc., all of which have been confirmed in other studies [22,23,24,25,26]. Our extensive questionnaire, with well identified factors, has made it possible to collect all the causes and reasons that lead nurses to come to work or to stay at work even when they are not feeling well, thus allowing us to obtain a very complete view of the phenomenon, which is useful for carrying out policies of care and attention to nurses [1,2,3].

Limitations. This was a cross-sectional study through an online questionnaire, which has resulted in a moderate sample size and the results obtained should be treated with caution. Nevertheless, the main objective of the study was the creation and validation of a questionnaire, something that has been more than achieved. A future line of research will be to obtain a larger and representative sample of our region or of the whole country.

This implies a less optimal performance and, in some professions, it can mean errors are committed which result in serious consequences, as confirmed by the studies [1,3,8]. It is important to note that health professionals are an occupational group working with a sensitive population, caring for people who may be healthy or ill, and the quality of the care provided may depend on their work [9,10,11,12,13].

An appropriate evaluation of presenteeism in these collectives, in which nursing professionals stand out as representing the largest professional group, may place management teams in a better position to implement appropriate measures to prevent this practice and to counter the effects of the same [16,17,18,19,20]. Previous studies have already indicated that nurses are one of the professions with the highest rates of presenteeism, which can result in important consequences, especially for users [1,3,5]. Organizations should consider the role of sick leave policies on presenteeism and make changes to encourage employees to take sick leave or stay away from work if they are sick, thereby reducing the rate of presenteeism and its consequences [32].

This study opens a new line of research for further studies on presenteeism in nurses in Spain, based on the development of the validated questionnaire presented herein.

## 5. Conclusions

The present study has created and validated a specific and well-structured question-naire to comprehensively assess the reasons for presenteeism among nurses. We have also explored the prevalence of presenteeism and the characteristics of nurses who have come or come to work sick. We can conclude that having a valid and adequate tool to study this phenomenon is important for making health management decisions. We know that nurs-es are the healthcare group with the most presenteeism, with serious consequences for their own health and for patient safety. Caring for professionals, something clearly stated in times of pandemic by COVID-19, is fundamental to ensure the sustainability of the healthcare system and guarantee quality care.

## Figures and Tables

**Table 1:** Commonalities.

Item	Extraction
Number of times	0.96
Performance	0.87
Adverse effects	0.71
Superiors	0.51
Colleagues	0.67
Sick leave	0.81
Less faculties	0.83
Reason	0.95
Pathologies	0.92

**Table 2:** Total explained variance.

Component	Rotation of Sums of Squared Loading		
	Total	% Variance	% Accumulated
1	3.22	35.84	35.84
2	2.29	25.51	61.32
3	1.73	19.32	80.63

**Table 3:** Results of the exploratory factorial analysis and factorial saturation of the items after the Varimax rotation.

Item	Factor		
	1	2	3
Sick leave	**0.875**	0.164	−0.147
Reason	**0.935**	−0.194	0.205
Pathologies	**0.905**	−0.226	0.225
Performance	−0.241	**0.880**	−0.203
Superiors	0.024	**0.712**	0.060
Colleagues	−0.078	**0.816**	−0.035
Decreased faculties	0.113	0.595	**0.687**
Number of times	−0.084	−0.039	**0.978**
Adverse effects	0.280	−0.181	**0.774**
Extraction method: analysis of main components. Rotation method: Varimax with Kaiser normalization.			

Bold type, the maximum value of saturation in the factor.

**Table 4:** Main sociodemographic and work-related variables.

Variable	Percentage	Variable	Percentage
Age (M/SD)	41.52 (+/− 11.74)	Do you work? % (*n* = 352)	
		Yes	98.3
		No	1.7
Sex	87.2% female	Type of center (*n* = 355)	
	12.8% male	Public	89.5
		Private	9.7
Marital status (*n* = 352)		Type of institution (*n* = 355)	
Single	45.2	Public Hospital	69.9
Married	48	Primary care	14.2
Separated	0.9	SUAP	2.6
Divorced	2.3	Private hospital	3.4
Widow/widower	1.4	Socio-sanitary	4
No reply	2.3	Preventive care	2
		Insurance companies	1.1
		TEA	0.3
		Others	2
		Public + Private hospital	0.3
		Public hospital + preventive care	0.3
Number of employees in the company (*n* = 352)			
Less than 10		Service	
10–19	0.6	Hospitalization	26.2
20–49	1.1	Consultations	5.8
50–99	6.5	Special services	54.2
100–249	2.3	Emergency services	2.2
250–499	5.4	Others	11.6
500–999	2.3		
Over 1000	5.4		
	76.4		
Academic title (*n* = 352)			
ATS		Work hours	
DipN	0.6	Reduced	3.4
DipN + BS	63.6	Fixed shifts	45.7
BS	19.3	Rotating shift	43.8
MSc	3.1	Split shift	1.1
PhD	2.6	Intensive	6
DipN + MSc	0.6		
DipN + BS + MSc	3.4		
BSc + MSc	5.1		
ATS + DUE	0.3		
Experience (*n* = 352)	1.4		
Less than 1 year		Type of contract	
Between 1–5 years	3.7		
Between 11–15	29.5	Indefinite	52.1
Between 16–20	17.3	Fixed-term	23.8
Over 20	10.5	Interim	24.1
Specialty (*n* = 352)	38.9		
Midwife		Multiple employment	
Mental health	3.1	Yes	9.7
Geriatrics	1.7	No	90.3
Family	2.3		
Pediatrics	8.8		
None	1.7		
Mental + Family Health	1.1		
	80.7		

ATS: Ayudante Técnico Sanitario, former title of nursing degrees in Spain, equivalent to a Diploma in Nursing; BS: Bachelor of Science; DipN: Diploma in Nursing; MSc: Master of Science. SUAP: Primary care emergency services; TEA: Temporary Employment Agency.

## Data Availability

Not applicable.
